# REGULATORY PROFILES AND POTENTIALLY BURDENSOME TRANSITIONS AMONG ASSISTED LIVING DECEDENTS

**DOI:** 10.1093/geroni/igac059.758

**Published:** 2022-12-20

**Authors:** Xiao (Joyce) Wang, Lindsey Smith, Joan Teno, Nicole Rosendaal, David Dosa, Pedro Gozalo, Kali Thomas, Emma Belanger

**Affiliations:** Brown University, Providence, Rhode Island, United States; Brown University, Providence, Rhode Island, United States; Brown University, Providence, Rhode Island, United States; Brown University, Providence, Rhode Island, United States; Brown University, Providence, Rhode Island, United States; Brown University, Providence, Rhode Island, United States; Brown University, Providence, Rhode Island, United States; Brown University, Providence, Rhode Island, United States

## Abstract

Potentially burdensome transitions at the end of life (i.e. repeated hospitalizations or transitions in the last 3 days of life) are common among assisted living (AL) residents, and are associated with lower care satisfaction by family members. AL regulations vary widely within and between states. This study aimed to describe the rate of burdensome transitions by AL regulatory profile, using a retrospective cohort study combining state AL regulations and multiple administrative claims data. The sample included 4,911 ALs serving 67,319 residents, who 1) died between 2017 and 2019; 2) resided in AL 120 days before death; 3) were continuously enrolled as fee-for-service one-year before death; and 4) resided in ALs with 25+ beds. The independent variable was a categorical variable indicating AL regulatory profile (i.e. housing, hybrid, hospitality, healthcare, rebalancing, hybrid healthcare), identified with a previously published methodology. These profiles differ in the allowance of third-party services, skilled nursing, medication administration, and requirements for medication review and licensed nursing staff. We first ran regression models to estimate the rate of burdensome transitions, accounting for AL-level resident demographic, socioeconomic, case-mix, and market characteristics. Results showed ‘hybrid’ ALs (i.e. low prevalence of skilled nursing allowed) had the highest rate of burdensome transitions (24.9%), whereas ‘healthcare’ ALs (i.e. high specificity for all types of services) had the lowest rate (22.2%). The rate of potentially burdensome transitions did not differ much by AL regulatory types, requiring more work to determine the drivers for burdensome transitions beyond AL regulations.

